# Community pharmacist-administered injectable naltrexone for individuals who were formerly incarcerated: a review of Wisconsin legislation and regulations

**DOI:** 10.1186/s13011-025-00647-9

**Published:** 2025-04-05

**Authors:** Jason S. Chladek, Michelle A. Chui

**Affiliations:** 1 Grossman School of Medicine, New York University, 180 Madison Ave, New York, NY 10016 USA; 2 Division of Social and Administrative Sciences, University of Wisconsin-Madison School of Pharmacy, 777 Highland Ave, Madison, WI 53705 USA; 3 Sonderegger Research Center, University of Wisconsin-Madison School of Pharmacy, 777 Highland Ave, Madison, WI 53705 USA

**Keywords:** Community pharmacists, Injectable naltrexone, Formerly incarcerated, Wisconsin, Legislation, Statutes, Regulations, Administrative codes, Law

## Abstract

Opioid use disorder (OUD) is highly prevalent among jail and prison populations in the United States, including in Wisconsin. Medications for opioid use disorder (MOUD), including long-acting injectable naltrexone, are crucial in the treatment of OUD. These medications are especially important for individuals transitioning out of jail or prison and back into the community. Unfortunately, few individuals who were formerly incarcerated continue MOUD after reentry due to a variety of barriers. Wisconsin community pharmacists are highly accessible and uniquely positioned to provide care for this population, specifically by administering injectable naltrexone. However, community pharmacist-administered injectable naltrexone for individuals who were formerly incarcerated has not been previously explored. As a first step, this legislative and regulatory review aimed to identify Wisconsin statutes and administrative codes that may impact these services for this population. Two legal databases were searched to identify relevant Wisconsin statute and administrative code subsections. Overall, 24 statute subsections (from 7 chapters) and 31 administrative code subsections (from 12 chapters) were identified that (1) highlighted a need or potential role of community pharmacist-administered injectable naltrexone for individuals who were formerly incarcerated or (2) served as a potential barrier or facilitator to the availability, access, or use of these services. Future work should focus on helping community pharmacists leverage available resources and overcome existing legal barriers to providing or supporting MOUD services. Importantly, work should be done to ensure that individuals who were formerly incarcerated can be linked to these services upon reentry.

**Supplementary Information:**

The online version contains supplementary material available at 10.1186/s13011-025-00647-9.

## Introduction

The opioid crisis is a major public health issue and has led to more than three million United States citizens suffering from opioid use disorder (OUD), a specific substance use disorder (SUD) characterized by a pattern of continued opioid use despite harmful health and social outcomes [1–3]. Wisconsin has been hard-hit by this crisis, as there were 1,464 opioid-related deaths in 2022 alone [4]. Opioid use disorder is highly prevalent among individuals impacted by the legal system, including in Wisconsin. Notably, the Wisconsin Department of Corrections (DOC) reported an 89% increase in overdose hospitalizations among individuals who were formerly incarcerated from 2013 to 2019. The Department also reported a 49% increase in overdose deaths among this same population from 2014 to 2020 [5]. 

Medications for opioid use disorder (MOUD), including long-acting injectable naltrexone, are a crucial component of OUD treatment [6]. As a result, several carceral facilities have started providing MOUD due to the high prevalence of OUD among individuals within the facilities, though there is still progress to be made [7]. Beyond being available within carceral facilities, MOUD is especially important for individuals transitioning out of these facilities and back into the community. Those receiving MOUD are less likely to have a drug overdose and/or face rearrest after release [8]. Unfortunately, few individuals who were formerly incarcerated continue MOUD upon community reentry. Previous research has shown that less than half of these individuals who are released with doses of MOUD continue to use it in the community [9–12]. As a result, this population has incurred a 40-fold greater likelihood of opioid overdose following reentry compared to the general population, and 40–50% of these individuals are arrested for a new crime within a year of release. (13–14)

Individuals who were formerly incarcerated face several barriers to accessing MOUD within the community. For instance, factors such as unstable housing, lack of insurance, lack of transportation, and drug cost can deter individuals from continuing treatment. Individuals may also be inhibited by a lack of motivation or fear of stigma with using MOUD. These individuals may also lack social support, have networks with limited treatment knowledge, or reside in areas with few or inflexible treatment resources [15–26]. Furthermore, carceral facilities do not always facilitate community linkage to MOUD when these individuals reenter the community. For example, in Wisconsin, less than half of jails provided individuals who were formerly incarcerated with resources for accessing MOUD upon reentry [9]. 

In general, research focused on improving access to MOUD within the community for individuals who were formerly incarcerated remains limited [27]. One resource that has not been previously explored is community pharmacists. In Wisconsin, community pharmacists can administer injectable naltrexone, an effective and widely accepted MOUD option for individuals impacted by the legal system and the most prevalent option within Wisconsin jails and prisons. (9, 28–29) Importantly, the success of interventions such as mobile treatment show that accessible MOUD locations and flexible treatment options can facilitate MOUD uptake by individuals who were formerly incarcerated within the community [30]. Previous work has shown that community pharmacies offer an accessible and flexible location for patients to seek care. (31–32) This can be especially important for those without stable housing or transportation. Furthermore, community pharmacists have knowledge on the pharmacological aspects of MOUD and can add to the available resources within a community [33]. 

In Wisconsin, there is a clear need to improve access to MOUD for individuals who were formerly incarcerated and are reentering the community, and community pharmacists are uniquely positioned to help address this need. However, community pharmacist-administered injectable naltrexone for individuals who were formerly incarcerated has not been previously explored. Accordingly, the objective of this study is to explore the legal environment in Wisconsin and identify legislation and regulations that potentially impact community pharmacist-administered injectable naltrexone for individuals who were formerly incarcerated.

## Methods

A legislative and regulatory review was conducted to compile and synthesize the existing statutes and administrative codes in Wisconsin with implications for community pharmacist-administered injectable naltrexone for individuals who were formerly incarcerated within the community. Statutes include acts passed by the Wisconsin Legislature. Administrative codes include regulations made by state executive agencies to guide how these agencies enforce statutes [34]. 

The lead researcher (JC) met with three librarians at the University of Wisconsin-Madison Ebling Library and Law School to discuss optimal search methods for identifying relevant statutes and codes. A scoping review approach was used as a guiding framework for this review [35]. Similar methodology was used in previous research analyzing laws related to pharmacist-provided MOUD services [28]. The librarians assisted the researcher in identifying keywords to use in the initial search. Keywords were categorized using an adapted version of the Patient/Population, Intervention, Comparison, and Outcome (PICO) method and are outlined in Table 1 [36]. An adapted version of PICO was utilized to better fit the study objective, as this study did not seek to compare outcomes between different groups. The lead researcher (JC) then used these keywords to search relevant electronic databases, including Fastcase and Thomas Reuters Westlaw. The search string is further detailed in Supplementary Material Section 1.

**Table 1:** Keywords used in initial database search

Population	Condition	Intervention	Environment
• Prison	• Opioid use disorder	• Medication	• Transition
• Jail	• Substance use disorder	• Medications for opioid use disorder	• Reentry
• Criminal	• Drug addiction	• Medication-assisted treatment	• Community
• Correction	• Drug abuse	• Substance use disorder treatment	
• Justice	• Substance abuse	• Substance abuse treatment	
• Incarcerated		• Methadone	
• Incarceration		• Buprenorphine	
		• Naltrexone	
		• Pharmacy	
		• Pharmacist	

The initial search was used to identify Wisconsin statute and administrative code chapters that included the keywords in Table 1. Full chapters were compiled, and no duplicates were included between the two databases. In total, 26 statute chapters and 40 administrative code chapters were included for full review. To identify relevant subsections, the researchers determined two a priori categories related to the study objective, including subsections that (1) highlighted a need or potential role of community pharmacist-administered injectable naltrexone for individuals who were formerly incarcerated or (2) served as a potential barrier or facilitator to the availability, access, or use of these services. The lead researcher (JC) manually reviewed full chapters for specific subsections that fit these categories. Ambiguities and/or questions were addressed in meetings between both researchers. After the manual review, 24 statute subsections (from 7 chapters) and 31 administrative code subsections (from 12 chapters) were included and summarized in table format. Both researchers then met to discuss implications for each subsection. All statutes and administrative codes were current as of July 2024.

## Results

Overall, 7 Wisconsin statute chapters included subsections with implications for community pharmacist-administered injectable naltrexone services for individuals who were formerly incarcerated [37–43]. These 7 chapters are outlined in Table 2. From these 7 chapters, a total of 24 relevant subsections were identified. Additionally, 12 Wisconsin administrative code chapters included subsections with implications for community pharmacist-administered injectable naltrexone services for individuals who were formerly incarcerated [44–55]. These 12 chapters are outlined in Table 3. From these 12 chapters, a total of 31 relevant subsections were identified. All subsections and potential implications are summarized below.

**Table 2:** Statute chapters with implications for community pharmacist-administered injectable naltrexone for individuals who were formerly incarcerated [37–43]

Chapter numbers and titles	
• Chapter 46: Social services • Chapter 49: Public assistance and children and family services • Chapter 51: State alcohol, drug abuse, developmental disabilities and mental health act • Chapter 146: Miscellaneous health provisions • Chapter 153: Health care information • Chapter 301: Corrections • Chapter 450: Pharmacy examining board	

**Table 3:** Administrative code chapters with implications for community pharmacist-administered injectable naltrexone for individuals who were formerly incarcerated [44–55]

Chapter numbers and titles	
• Department of Corrections (DOC) Chap. 302: Inmate classification, sentence, and release provisions • Department of Corrections (DOC) Chap. 333: Incentive sanctions • Department of Children and Families (DCF) Chap. 105: Substance Abuse Screening, Testing and Treatment for Certain Department Work Experience Programs • Department of Health Services (DHS) Chap. 36: Comprehensive community services for persons with mental disorders and substance-use disorders • Department of Health Services (DHS) Chap. 38: Substance Abuse Screening, Testing and Treatment for Certain Department Employment and Training Programs • Department of Health Services (DHS) Chap. 66: Treatment Alternative Program • Department of Health Services (DHS) Chap. 75: Community Substance Use Services Standards • Department of Health Services (DHS) Chap. 83: Community-based residential facilities • Department of Health Services (DHS) Chap. 107: Covered services • Office of the Commissioner of Insurance (Ins) Chap. 8: Employee Welfare Funds; Employee Benefit Plan Administrators; Small Employer Health Insurance • Pharmacy Examining Board (Phar) Chap. 7: Pharmacy practice • Pharmacy Examining Board (Phar) Chap. 16: Continuing education for pharmacists	

### Need and potential roles of community pharmacist-administered injectable naltrexone

From the statute and administrative code subsections that were identified in the review, several emphasized the need for MOUD providers and/or injectors for individuals who were formerly incarcerated, a role that community pharmacists could help fill. There are several state-level programs and facilities, such as treatment alternative programs (TAPs), Comprehensive Community Services (CCS) programs, work programs, and recovery residencies, that can be utilized by individuals impacted by the legal system, including individuals who were formerly incarcerated. These programs and facilities recommend or even require that individuals receive treatment for SUDs, which may include MOUD.

Additionally, there are several collaborative roles that community pharmacists could take. The identified subsections highlight various departments, groups, and agencies, such as the Milwaukee County Mental Health Board or Council of Offender Reentry, that support individuals who were impacted by the legal system, formerly incarcerated, and/or dealing with SUDs. Through fostering collaborative relationships, community pharmacists could potentially work with these groups to increase access to MOUD resources, specifically by administering injectable naltrexone to individuals needing this treatment option. Community pharmacists could also increase collaborations with MOUD providers and take on the role of administering injectable naltrexone for their patients, including those who were involved with the legal system or formerly incarcerated. All subsections related to the need or potential roles of community pharmacist-administered injectable naltrexone for individuals who were formerly incarcerated are included in Table 4.

**Table 4:** Subsections highlighting the need and potential roles of community pharmacist-administered injectable naltrexone for individuals who were formerly incarcerated

Subsections of statute chapters	
**Chapter 46: Social services** [37]	
46.234(2)(c) The department shall maintain a registry of approved recovery residencies, and all residencies must agree to help facilitate active recovery for residents. 46.234(3) The department may not include a recovery residence in the registry if it excludes a resident from participating in medication-assisted treatment. 46.482(2–4) The department shall maintain a program to facilitate overdose treatment providers to help coordinate and continue care and treatment of individuals after an overdose, including referral to treatment services. Care coordination may include transportation to and from treatment. The department may seek grant funding to establish and maintain the program. 46.65(1) The department shall implement a treatment alternative program. The department shall make grants to provide alcohol or other drug abuse services, as a treatment alternative in lieu of imprisonment, for eligible persons in need of those services. The department shall make grants so that the treatment alternative program serves a variety of geographic locations. 46.973(2) A drug dependence and drug abuse program is established in the department. The secretary may develop and carry out programs concerned with education about and prevention of drug dependence and drug abuse and programs concerned with treatment and rehabilitation of drug dependent persons and persons who abuse drugs.	
**Chapter 51: State alcohol**,** drug abuse**,** developmental disabilities and mental health act** [39]	
51.41(1s) The Milwaukee County Mental Health Board shall facilitate delivery of mental health services in an efficient and effective manner by making a commitment to community-based, person-centered, recovery-oriented, mental health systems and maximizing comprehensive community-based services. 51.448(1–3) The department shall create and administer an addiction medicine consultation program to assist participating clinicians (physicians, nurse practitioners, and physician assistants) in providing enhanced care to patients with substance use addiction and to provide referral support for patients with a substance abuse disorder. The department shall review proposals and provide money to organizations seeking to provide consultation services through the addiction medicine consultation program under this section. 51.45(4) The department shall develop, encourage and foster statewide, regional, and local plans and programs for the prevention of alcoholism and drug dependence and treatment of alcoholics, persons who are drug dependent, and intoxicated persons in cooperation with public and private agencies, organizations, and individuals and provide technical assistance and consultation services for these purposes. In doing so, the department shall coordinate with all public and private agencies, organizations and individuals interested in prevention of alcoholism and drug dependence and treatment of alcoholics, persons who are drug dependent, and intoxicated persons. 51.45(7)(a-c) The department shall establish a comprehensive and coordinated program for the treatment of alcoholics, persons who are drug dependent, and intoxicated persons. The program of the department shall include outpatient and follow-up treatment. The department shall provide for adequate and appropriate treatment for alcoholics, persons who are drug dependent, and intoxicated persons.	
**Chapter 146: Miscellaneous health provisions** [40]	
146.89(3)(a-b) Any volunteer health care provider and nonprofit agency whose joint application is approved may provide the following health care services: diagnostic tests, health education, information about available health care resources, office visits, patient advocacy, prescriptions, and referrals to health care specialists.	
**Chapter 301: Corrections** [42]	
301.068(1–2) The department shall establish community services that have the goals of increasing public safety, reducing the risk that offenders on community supervision will reoffend, and reducing recidivism rates. In establishing community services, the department shall consider the capacity of existing services and any needs that are not met by existing services. The community services to reduce recidivism shall include: (1) alcohol and other drug treatment, including residential treatment, outpatient treatment, and aftercare and (2) treatment and services that evidence has shown to be successful and to reduce recidivism. 301.095 The Council on Offender Reentry shall do all of the following: (1) coordinate reentry initiatives and research federal grant opportunities, (2) identify methods to improve collaboration and coordination of offender transition services, (3) identify funding opportunities to maximize the use of state and community-based services as the services relate to reentry, (4) identify and review existing reentry policies, programs, and procedures, (5) promote collaboration and communication between the department and community organizations that work in offender reentry, (6) work to include victims in the reentry process and promote services for victims while the offenders are incarcerated and after the offenders are released.	
**Chapter 450: Pharmacy examining board** [43]	
450.033 A pharmacist may perform any patient care service delegated to the pharmacist by a physician.	
**Subsections of administrative code chapters**	
**Chapter 302: Inmate classification**,** sentence**,** and release provisions**^44^]	
302.34(7) If the special action release (SAR) is granted, the secretary may impose in writing any special conditions that are appropriate. The conditions that the secretary may impose include requirements for outpatient treatment, including treatment for alcohol abuse or other drug abuse. 302.35(3) The department shall consider all of the following when making a decision to release an inmate under this section: (1) the inmate can complete programming and treatment in the community without presenting undue risk and (2) the inmate has developed an adequate release plan.	
**Chapter 333: Incentive sanctions** [45]	
333.05(2) Division of intensive sanctions (DIS) staff shall explain to the inmate the DIS rules of supervision and describe the treatment and services available, including mental health outpatient treatment and services and alcohol or other drug abuse outpatient treatment and services.	
**Chapter 105: Substance Abuse Screening**,** Testing and Treatment for Certain Department Work Experience Programs** [46]	
105.06(1–2) Every individual who tests positive for the use of a controlled substance without presenting evidence of a valid prescription shall be required to participate in treatment in order to be eligible to participate in a work experience program. The administering agency shall provide information to every individual required to participate in controlled substance abuse treatment about treatment program providers.	
**Chapter 36: Comprehensive community services for persons with mental disorders and substance-use disorders** [47]	
36.07(3) Each comprehensive community services (CCS) program shall have a written plan that shall include description of the currently available mental health, substance-use disorder, crisis services, and other services in the county or tribe and how the CCS will interface and enhance these services. The description shall include policies and procedures for developing and implementing collaborative arrangements and interagency agreements. 36.17(4a) Psychosocial rehabilitation and treatment services shall be provided in the most natural and least restrictive manner and most integrated settings practicable consistent with current legal standards, be delivered with reasonable promptness, and build upon the natural supports available in the community.	
**Chapter 38: Substance Abuse Screening**,** Testing and Treatment for Certain Department and Employment and Training Programs** [48]	
38.06(1–2) Every individual that tests positive for the use of a controlled substance without presenting a valid prescription shall be required to participate in trauma-informed controlled substance abuse treatment to remain eligible to participate in an employment and training program.	
**Chapter 66: Treatment Alternative Program** [49]	
66.05 Each TAP agency shall negotiate a written agreement with local criminal justice system components to assure the effective and accountable operation of the local TAP and maintain necessary communications regarding potential clients referred from the criminal justice system. They shall also maintain working relationships and mutual agreements with treatment agencies to assure the availability of treatment agency options, effective client referrals and necessary tracking and monitoring activities. 66.08(2) Within 48 h after the assessment is completed, a client shall be referred to a treatment program. If a treatment placement is not immediately available, TAP staff shall monitor the client during the interim period.	
**Chapter 75: Community Substance Use Services Standards** [50]	
75.49 “Outpatient substance use treatment service” means a non-residential treatment service in which substance use treatment personnel provide screening, assessment, and treatment for substance use disorders. A service may provide outpatient substance use treatment services in the community or other locations, provided all requirements of this chapter are able to be met in the setting, the services has written policies and procedures, and the services provides annual training for all staff.	
**Chapter 83: Community-based residential facilities** [51]	
83.37(1)(e) If residents’ medications are administered by a community-based residential facility (CBRF) employee, the CBRF shall arrange for a pharmacist or a physician to review each resident’s medication regimen. This review shall occur within 30 days before or 30 days after the resident’s admission, whenever there is a significant change in medication, and at least every 12 months. At least annually, the CBRF shall have a physician, pharmacist, or registered nurse conduct an on-site review of the CBRF’s medication administration and medication storage systems. 83.37(1)(h) When a psychotropic medication is prescribed for a resident, the CBRF shall ensure the resident is reassessed by a pharmacist, practitioner or registered nurse at least quarterly. 83.37(1)(k) The CBRF shall report all errors in the administration of medication, any adverse drug reactions, and any resident who refuses a medication for two consecutive days to a licensed practitioner, supervising nurse or pharmacist immediately. 83.37(2)(b) When medication administration is supervised by a pharmacist, the CBRF shall ensure that the pharmacist coordinates, directs and inspects the administration of medications and the medication administration system, participates in the resident’s assessment and development and review of the individual service plan regarding medical conditions and goals of the medication regimen. 83.37(1)(h) The CBRF shall provide medication administration appropriate to the resident’s needs.	
**Chapter 107: Covered services** [52]	
107.10(5) The pharmacist shall review the drug therapy before each prescription is filled or delivered to an MA recipient and offer consultation.	
**Chapter 7: Pharmacy practice** [54]	
7.02(3)(a) A practitioner may transmit a prescription order electronically only if the patient approves the transmission and the prescription order is transmitted to a pharmacy designated by the patient. 7.03(1) A pharmacist shall complete a drug utilization review by reviewing the patient record prior to dispensing each prescription drug order.	

### Potential barriers or facilitators to community pharmacist-administered injectable naltrexone

In addition to the statute and administrative code subsections discussed above, several subsections also highlighted potential barriers or facilitators to the availability, access, or use of community pharmacist-administered injectable naltrexone by individuals who were formerly incarcerated. In general, the language used in many subsections either excludes or does not implicitly recognize pharmacists as a resource for MOUD. These subsections also highlight requirements for pharmacists to administer injectable naltrexone, including completing a training course and reviewing a comprehensive patient profile prior to dispensing or administering any medication. These requirements may inhibit community pharmacists from providing naltrexone injection services, including for individuals who were formerly incarcerated.

On the other hand, certain subsections may facilitate these services. Importantly, subsections of Chap. 450: Pharmacy examining board and Pharmacy Examining Board Chap. 7 provide the legal authority for community pharmacists to administer naltrexone injections for any patient with a valid prescription. Additionally, pharmacists can enter a collaborative practice agreement (CPA) with a physician, allowing the physician to delegate patient care services – including management of individuals with SUDs – to the pharmacist. Pharmacists are also recognized as providers under Medicaid, allowing them to be reimbursed for services within their scope of practice, including administering injectable naltrexone. Finally, numerous subsections highlight sources of state-level funding for SUD treatment that could potentially be pursued by community pharmacists to support injectable naltrexone services and help treat individuals who were formerly incarcerated. All subsections related to potential barriers and facilitators are listed in Table 5.

**Table 5:** Subsections highlighting potential barriers and facilitators to community pharmacist-administered injectable naltrexone for individuals who were formerly incarcerated

Subsections of statute chapters	
**Chapter 46: Social services** [37]	
46.40(1)(a) The department may distribute funds for community social, mental health, developmental disabilities, and alcohol and other drug abuse services. 46.47(1–3) The department may award grants to provide nonnarcotic, non-addictive, injectable medically assisted treatment to a county that has a jail or drug court, provides care coordination for reentering inmates, and has identified how it will ensure all program participants are enrolled in Medical Assistance and will continue treatment upon reentry. 46.48(30) The department may distribute grants to private nonprofit organizations for the provision of alcohol and other drug abuse treatment services in counties with a population of 750,000 or more. Treatment should only be provided to individuals who are eligible for federal temporary assistance for needy families. 46.975(2) The department shall allocate funds to community-based organizations for providing drug abuse interventions and treatment directed at low-income Hispanics and Black Americans in urban areas, women, and youth.	
**Chapter 49: Public assistance and children and family services** [38]	
49.167(1) The department may award grants private entities to provide community-based alcohol and other drug abuse treatment programs that are targeted at individuals who have a family income of not more than 200% of the poverty line and who are eligible for temporary assistance for needy families. 49.46(2)(bh) The department shall provide reimbursement for services that are reimbursable under this section and that are provided by a licensed pharmacist within the scope of his or her license.	
**Chapter 51: State alcohol**,** drug abuse**,** developmental disabilities and mental health act** [39]	
51.4224(1) “Opioid treatment system” means a structured delivery system for providing substance abuse prevention, intervention, or treatment services and 1) receives funds through the state under this chapter and 2) is approved by the state methadone authority. 51.423(1) The department shall fund, within the limits of the department’s allocation for mental health services, services for mental illness, developmental disability, alcoholism, and drug abuse to meet standards of service quality and accessibility.	
**Chapter 153: Health care information** [41]	
153.87 The department of administration shall issue a request for proposals to establish and maintain an opioid and methamphetamine data system to collect, format, analyze, and disseminate information on opioid and methamphetamine use, which shall include (1) the number of opioid treatment centers in the state and (2) the number of persons who are incarcerated, on extended supervision or probation, or on parole and who are receiving naltrexone for extended-release in injectable suspension.	
**Chapter 450: Pharmacy examining board** [43]	
450.035(1)(r) A pharmacist may not administer by injection a prescribed drug product or device unless he or she has successfully completed a course of study and training in administration technique conducted by a course provider approved by the Accreditation Council for Pharmacy Education or the board. 450.085(1) An applicant for renewal of a license shall submit proof that he or she has completed, within the 2-year period immediately preceding the date of his or her application, 30 h of continuing education in courses conducted by a provider that is approved by the Accreditation Council for Pharmacy Education or in courses approved by the board.	
**Subsections of administrative code chapters**	
**Chapter 105: Substance Abuse Screening**,** Testing and Treatment for Certain Department Work Experience Programs**[46]	
105.06(1–2) Every individual who tests positive for the use of a controlled substance without presenting evidence of a valid prescription shall be required to participate in treatment in order to be eligible to participate in a work experience program. The administering agency shall provide information to every individual required to participate in controlled substance abuse treatment about treatment program providers.	
**Chapter 36: Comprehensive community services for persons with mental disorders and substance-use disorders** [47]	
36.10(2 g) Each staff member (psychiatrists, physicians, psychologists, social workers, counselors, therapists, nurses, physician assistants, occupational therapists, peer specialist, and rehabilitation workers) shall have the interpersonal skills training and experience needed to perform the staff member’s assigned functions.	
**Chapter 66: Treatment Alternative Program** [49]	
66.01 The alcohol or other drug abuse (AODA) treatment alternative program (TAP) consists of grants made by the department to local agencies to provide TAP services, including assessment and treatment services, to persons likely to benefit from those services who are referred from courts, law enforcement agencies, probation and parole agents and other parts of the criminal justice system. 66.03(1) To be eligible for a TAP grant an agency shall be certified to operate one or more AODA programs under Chapter DHS 75.	
**Chapter 75: Community Substance Use Services Standards** [50]	
75.12 All requirements in this chapter shall also be applicable to telehealth services delivered under this chapter. 75.18 A service shall have a service director, clinical supervisor, substance abuse counselor, prescribers, nurses, and mental health professionals. 75.59(1–2, 10) “Opioid treatment program,” or “OTP,” means a service that provides for the management and rehabilitation of persons with an opioid use disorder through the use of FDA-approved medications. Additionally, OTPs shall provide adequate medical, counseling, vocational, educational, and other assessment and treatment services. For medical needs of a patient that exceed the scope of the service under this chapter, the service shall coordinate with appropriate medical providers.	
**Chapter 83: Community-based residential facilities** [51]	
83.37(2)(e) Injectables, nebulizers, stomal and enteral medications, and medications, treatments or preparations delivered vaginally or rectally shall be administered by a registered nurse or by a licensed practical nurse within the scope of their license. 83.38(1)(k) The CBRF shall provide or arrange transportation when needed for medical appointments, work, educational or training programs, religious services and for a reasonable number of community activities of interest.	
**Chapter 107: Covered services** [52]	
107.10(1) Drugs and drug products covered by Medical Assistance (MA) include legend and non-legend drugs and supplies listed in the Wisconsin Medicaid drug index which are prescribed by a physician, dentist, optometrist, advanced practice nurse, or when a physician delegates the prescribing of drugs to a nurse practitioner or to a physician’s assistant.	
**Chapter 8: Employee Welfare Funds; Employee Benefit Plan Administrators; Small Employee Health Insurance** [53]	
8.72(16) Each health benefit plan shall provide coverage for outpatient and transitional treatment for nervous and mental disorders and alcoholism and other drug abuse if medically necessary.	
**Chapter 7: Pharmacy practice** [54]	
7.13(1–4) A pharmacist may administer a drug product or device and, after administration, notify the prescribing practitioner or enter the information in a patient record system shared by the prescribing practitioner. A pharmacist may not administer an injectable drug product or device unless they have completed a course of study and training in administration technique conducted by a course provider approved by the Accreditation Council for Pharmacy Education or the board.	
**Chapter 16: Continuing education for pharmacists** [55]	
16.02(1) Each pharmacist shall sign a statement on the application for license renewal certifying that the pharmacist has completed at least 30 hours of acceptable continuing education programs within the 2-year period immediately preceding the date of his or her application for renewal. 16.03 The board recognizes only those educational programs offered by a provider approved by the Accreditation Council for Pharmacy Education at the time of attendance or other board approved programs.	

## Discussion

As shown, the legislative and regulatory review identified several Wisconsin statute and administrative code chapters and subsections with potential implications for community pharmacist-administered injectable naltrexone for individuals who were formerly incarcerated. The subsections both highlighted the need and potential roles for community pharmacist-administered injectable naltrexone for individuals who were formerly incarcerated and included potential barriers and facilitators to these services. Notably, the requirements of several state-level programs and residences often used by individuals who were formerly incarcerated emphasize a need for available treatment resources for those with SUDs, including MOUD providers and injectors. By administering injectable naltrexone, community pharmacists can add to the available resources and support this need. Additionally, community pharmacists may collaborate with several departments and agencies outlined in the legislation that support individuals who were formerly incarcerated and/or deal with SUDs. Some of these groups even have goals specifically related to supporting reentry. For example, the Council of Offender Reentry (as outlined in Wisconsin Statute Chap. 301: Corrections) serves several purposes, including coordinating reentry initiatives, identifying methods to improve coordination of transition services, and promoting collaboration between the department and community organizations [42]. Overall, fostering relationships between pharmacists and these groups can help improve awareness of community pharmacy services, facilitate pharmacist involvement, and increase referrals to community pharmacist-administered injectable naltrexone for those needing treatment.

The subsections in this review may also serve as barriers or facilitators to community pharmacist-administered injectable naltrexone for individuals who were formerly incarcerated. Those that do not included pharmacists in the language describing SUD treatment resources, as well as those outlining training and medication review requirements, may hinder awareness and/or availability of community pharmacist-administered injectable naltrexone. However, subsections outlining pharmacist scope of practice authority, reimbursement, and funding opportunities may facilitate the ability of community pharmacists to offer these services. Opportunities like CPAs can both support pharmacists’ abilities to provide patient care services (such as administering injectable naltrexone) and help pharmacists foster relationships with MOUD prescribers. It should also be noted that some subsections have mixed implications for community pharmacist-provided injectable naltrexone for individuals who were formerly incarcerated. For example, Wisconsin law allows providers to deliver care via telehealth. This can facilitate the use of community pharmacist-administered naltrexone injections, especially if individuals who were formerly incarcerated are able to obtain a prescription via telehealth. However, individuals who were formerly incarcerated may not have access to the resources required to attend a virtual healthcare visit (phone, computer, internet, etc.), which has been a concern in previous research [56]. Additionally, community pharmacists are required to complete 30 hours of continuing education every two years to maintain their licensure. Continuing education programs provide an opportunity to educate community pharmacists on injectable naltrexone services and/or caring for patients impacted by the legal system. However, only programs approved by the Accreditation Council for Pharmacy Education (ACPE) can count toward this requirement [55]. As a result, the ACPE should ensure that such training programs exist and are available to community pharmacists.

Outside of implications for community pharmacists, a few subsections may impact access to treatment for individual patients. As an example, Medicaid and small employer health plans provide coverage for treatments related to substance use. Additionally, several residential facilities (including community-based residential facilities) and state-level programs provide transportation services to support individuals seeking treatment for SUDs. These subsections can further improve the accessibility of community pharmacist-provided injectable naltrexone for individuals who were formerly incarcerated and may face financial barriers or lack reliable transportation. (15–16, 19)

While this review focused on injectable naltrexone, it may have implications for other forms of MOUD. Naltrexone is one of three main MOUD options, in addition to methadone and buprenorphine [6]. In Wisconsin, community pharmacists are authorized to dispense buprenorphine products for the treatment of OUD [54]. Additionally, current research has explored community pharmacist involvement in providing buprenorphine and supporting certified opioid treatment programs (OTPs) that provide methadone. (57–58) Many of the potential collaborations, barriers, and facilitators identified in this review could likely impact the ability of community pharmacists to support the use of other forms of MOUD for individuals who were formerly incarcerated. Additional work could be done to explore this further.

Additionally, although this review focused on Wisconsin legislation and regulations, the findings may also have implications for other states. For example, several other states have laws that provide pharmacists the legal authority to administer long-acting injectable medications, including injectable naltrexone [59]. Pharmacists in those states could explore the opportunity to support treatment for individuals who were formerly incarcerated, including via relationships with state-level departments, programs, or agencies that support this population. Work could also be done to explore legislative or regulatory barriers and facilitators in these states, such as training requirements, reimbursement for naltrexone injections, or potential funding sources. With that being said, there are certain areas where this review may be less applicable. As shown, in Wisconsin, many programs and groups that interact with individuals who were formerly incarcerated support the use of MOUD in treating OUD. However, in other states, similar programs and groups may not encourage MOUD for individuals who were formerly incarcerated or even promote abstinence-only approaches [60]. 

Lastly, it is clear that there are several programs and groups in Wisconsin that could collaborate with community pharmacists to increase the availability, access, and use of injectable naltrexone by individuals who were formerly incarcerated. However, as previously discussed, carceral facilities often fail to link individuals to MOUD resources within the community during reentry [9]. Ultimately, even if community pharmacists are collaborating with state or community-level groups that support individuals who were formerly incarcerated and need MOUD, it is likely that individuals still will not be referred to these services. Work needs to be done to not only ensure that there are enough MOUD resources within the community – a gap that community pharmacists can help fill – but that individuals reentering the community from carceral facilities are being linked to these resources. Ultimately, additional work should explore strategies for improving care linkage and coordination.

### Limitations

While this review was intended to be exploratory in nature, it presents a few limitations that should be acknowledged. First, it is possible that certain statutes and/or administrative codes were overlooked based on the search strategy or databases used. Additionally, for the purposes of this project, federal legislation and policies or guidelines from professional organizations were not included. This may have provided additional implications for the availability, access, and use of community pharmacist-administered injectable naltrexone for individuals who were formerly incarcerated in Wisconsin. The review was also focused on Wisconsin legislation and regulations, limiting the overall generalizability for other states or countries, especially those with different legislative landscapes. Also, while community pharmacists can administer injectable naltrexone, individuals still need an active prescription to receive the injection. Unless under a CPA, Wisconsin pharmacists cannot add new prescriptions. This review may not include certain statutes or administrative codes that impact the availability of MOUD prescribers or ability of individuals who were formerly incarcerated to obtain a naltrexone prescription prior to receiving an injection. Finally, while the review highlighted potential collaborations and funding opportunities for community pharmacy-administered injectable naltrexone for individuals who were formerly incarcerated, it does not explore whether or not community pharmacists are prioritizing or pursuing these opportunities. Overall, these limitations provide directions for future work.

## Conclusions

The legislative and regulatory review further emphasized the importance of increasing the number of available injectable naltrexone providers and injectors within Wisconsin, especially for individuals impacted by the legal system and those who were formerly incarcerated. Not only do community pharmacists have the authority to administer naltrexone injections, but there are several potential collaborations and funding opportunities that may help facilitate these services. It is important that future work focuses on helping community pharmacists leverage these resources, as well as overcome barriers identified in the legislation and regulations. Importantly, work should be done to ensure that individuals who were formerly incarcerated are connected to MOUD services, including pharmacist-administered injectable naltrexone. This work can help ensure that individuals who were formerly incarcerated are able to continue crucial medications and succeed after community reentry.

## Electronic supplementary material

Below is the link to the electronic supplementary material.

Supplementary Material 1

## Data Availability

No datasets were generated or analysed during the current study.

## Abbreviations

OUD: Opioid use disorder
SUD: Substance use disorder
DOC: Department of Corrections
MOUD: Medications for opioid use disorder
PICO: Patient/Population, Intervention, Comparison, and Outcome
DCF: Department of Children and Families
DHS: Department of Health Services
SAR: Special action release
DIS: Division of Intensive Sanctions
CCS: Comprehensive Community Services
TAP: Treatment alternative program
CBRF: Community-based residential facility
AODA: Alcohol or other drug abuse
OTP: Opioid treatment program
CPA: Collaborative practice agreement
ACPE: Accreditation Council for Pharmacy Education
