# Supplementary material for: Community pharmacist-administered injectable naltrexone for individuals who were formerly incarcerated: a review of Wisconsin legislation and regulations
Source: Subst Abuse Treat Prev Policy. 2025 Apr 5;20:17. doi: 10.1186/s13011-025-00647-9 (PMC11972521; doi:10.1186/s13011-025-00647-9)
Supplement: Supplementary file 1 — Supplementary Material 1 [file 13011_2025_647_MOESM1_ESM.docx]

**Supplementary Material**

**Section 1: Search string used for all databases**

(prison OR jail OR criminal OR correction OR justice OR incarcerated OR incarceration) OR (opioid use disorder OR substance use disorder OR drug addiction OR drug abuse OR substance abuse) OR (medication OR medications for opioid use disorder OR medication-assisted treatment OR substance use disorder treatment OR substance abuse treatment OR methadone OR buprenorphine OR naltrexone OR pharmacy OR pharmacist) OR (transition OR reentry OR community)
